# Uncertainty and Motivation to Seek Information from Pharmacy Automated Communications

**DOI:** 10.3390/pharmacy6020047

**Published:** 2018-05-28

**Authors:** Michelle Bones, Martin Nunlee

**Affiliations:** 1 Department of Veterans Affairs, Voluntary Service, 1601 Kirkwood Highway, Wilmington, DE 19805 USA; mcnunlef@umich.edu; 2 Department of Business Administration, College of Business, Delaware State University, 1200 North Dupont Highway, Dover, DE 19901-2277, USA

**Keywords:** pharmacy, patient communication, pharmacy communications, interpersonal communications, automated telemarketing telephone calls, telephone messages, automated messages, communication theory, customer relation management, CRM, pharmacy practice

## Abstract

Pharmacy personnel often answer telephones to respond to pharmacy customers (subjects) who received messages from automated systems. This research examines the communication process in terms of how users interact and engage with pharmacies after receiving automated messages. No study has directly addressed automated telephone calls and subjects’ interactions. The purpose of this study is to test the interpersonal communication (IC) process of uncertainty in subjects in receipt of automated telephone calls ATCs from pharmacies. Subjects completed a survey of validated scales for Satisfaction (S); Relevance (R); Quality (Q); Need for Cognitive Closure (NFC). Relationships between S, R, Q, NFC, and subject preference to ATCs were analyzed to determine whether subjects contacting pharmacies display information seeking behavior. Results demonstrated that seeking information occurs if subjects: are dissatisfied with the content of the ATC; perceive that the Q of ATC is high and like receiving the ATC, or have a high NFC and do not like receiving ATCs. Other interactions presented complexities amongst uncertainty and tolerance of NFC within the IC process.

## 1. Introduction

Automated messaging is a major form of patient communication for community pharmacies. Voicemails, text messages or emails serve to notify patients. These forms of communication are by their nature unidirectional, from the pharmacy to the patient. For there to be bidirectional communication, the patient must contact the pharmacy—usually this contact is by telephone. How many times have personnel in pharmacies had to respond to customers who have called concerning communications from automated systems? Automated messages—specifically automated telephone messages dates back to 1924 [1]. Accordingly, the sentiments of the intent of the sender’s message, “… was believed…to save considerable expense to the companies where many “repeat” calls are necessary” [1]. The use of recorded messages blossomed in the late 20th century and exploded in the 21st century [2]. Often pharmacies send automated messages via telephone to patients, as a form of communication. The telephone, as a medium, is “cool” or one of low definition [3]. Automated telephone calls from pharmacies provide information requiring so much to be filled in by the pharmacy customer. When a pharmacy customer responds to the automated telephone call (ATC) from a pharmacy, the medium requires the individual to “actively analyze and interpret what is presented, to make sense of what they … hear” [4]. After receiving a message from a cool medium such as an ATC, the pharmacy customer can choose to respond to the message, thereby engaging in the interpersonal communication process. An alternative option is not to respond. 

**Figure 1:**
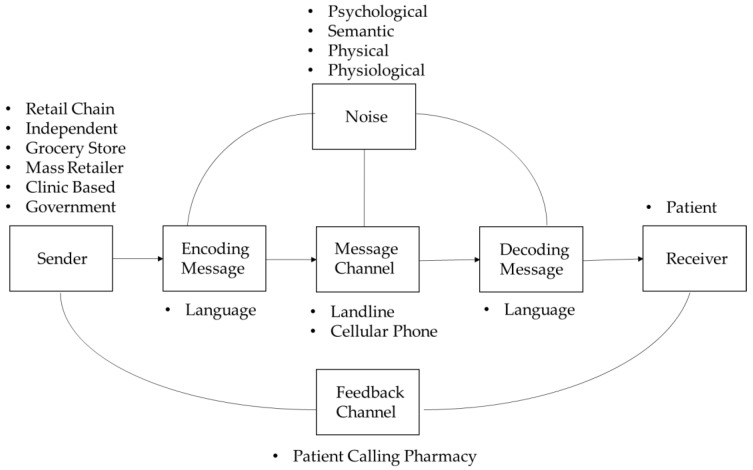
Diagram of communication process.

Very few studies directly addressed the role communication plays in pharmacy patients’ interactions; this is one of the few studies in the pharmacy discipline that seeks to dive in-depth into the communication and interpersonal communication concepts [5,6]. By definition communication is the process of imparting or interchanging thoughts, opinions or information. Fields of study as disperse as psychology, business and engineering rely upon the same basic model of communication. A diagram of the communications process is outlined in Figure 1. 

Figure 1 depicts the typical communication process. In this research we are concerned with how noise, specifically—psychological noise, influences patients’ perceptions within the domain of ATCs. As indicated in the figure, noise can impact the encoding and decoding, and the message channel. Noise is anything that hinders the communication process. The four most common forms of noise are physical, physiological, semantic, and psychological. Physical noise is something that interferes with communication that is external to both the sender and receiver. Physiological noise is an internal condition of the receiver or sender that causes a distraction. Physiological noise could be caused by hunger, fatigue, malaise, medication or other factors that affect how people feel and think. In terms of oral exchanges these internal distraction, may cause senders to have problem in articulation, while receivers may have problem in hearing. Semantic noise is primarily cause by the sender. It occurs when senders encode messages in language in which the receiver is unfamiliar. Most of the work concerning the communication process in healthcare has focused on semantic noise—in the form of patient health literacy. In its most basic form, psychological noise consists of mental interference. The reader should understand that psychological noise could stem from wandering thoughts and preconceived ideas; as well as dislike of the sender, medium or receiver. To measure psychological noise, we use common constructs and in-turn scales from psychology and consumer behavior to assess patients’ tendencies and attitudes and then gauge their response, by the number of feedback calls. 

The motivation for why people would contact pharmacies varies. Is the ATC a channel for information seeking by pharmacy patients? Will pharmacy customers call the pharmacy seeking information pertaining to the ATC? According to Brasher et al. [6], within the health context framework, information is defined as stimuli from a person’s environment that contribute to his or her knowledge or belief. Some customers will call to get clarification or confirmation, while other customers will call if they find the ATC from a pharmacy a bother or nuisance. What are the thoughts of the pharmacy patient following the receipt of an ATC from a pharmacy? If a pharmacy patient seeks information from the pharmacy, what are the contributions to the patient? Within the health context framework, “the information can be used to decrease uncertainty that is distressing, to increase certainty that allows for hope or optimism, and to invite reappraisal of uncertainty” [6]. Information seeking has been studied in the context of individuals’ network of interpersonal relationships. Theories from the Social Science discipline of Interpersonal Communication will serve as the basis of analysis for the pharmacy-pharmacy patient interpersonal relationship. The selected perspective is represented by the arc of “noise” to “decoding message” outlined in Figure 1. The content of the message is not a specific focus. However, the medium of the message, via telephone, contributes to the knowledge or beliefs of the pharmacy patient. Before, discussing motivation there has to be a clear understanding of what is meant by communication.

What is communication? “A process as complex as communication is hard to summarize or define” [7]; from a business marketing perspective, “communication is the process by which we exchange or share meanings through a common set of symbols” [8]. The highest level of communication can be further delineated, from broadcasting one-way messages to interactive interpersonal communication. Focusing on interpersonal communication, it is direct, face-to-face communication between two or more people [8], and the participants receive maximum feedback [7]. A further delineation of interpersonal communication is the theory of uncertainty. Brashers [9] states that “uncertainty exist when details of situations are ambiguous, complex, unpredictable, or probabilistic; when information is unavailable or inconsistent; and when people feel insecure in their own state of knowledge or the state of knowledge in general”. What factors, in an interpersonal communication relationship, can affect uncertainty? Need for Closure (NFC) is one factor. NFC is commonly defined “within the relationship exchange of information, [as] the desire for an answer on a given topic, any answer, as compared to confusion or ambiguity” [10]. 

Excluding pharmacy, “disciplines that have examined this depth of the uncertainty process in earnest include communications, psychology, sociology, family studies, library and information sciences, medicine, genetic counseling, business, economics and religious studies” [11]. Theories from the Social Science discipline of Interpersonal Communication serve as the basis of analysis for the pharmacy-pharmacy patient interpersonal relationship. To advance our understanding of the pharmacy-pharmacy patient communication process more empirical study is necessary. Further, socio-demographic characteristics should be examined within the framework of the information exchange process. This research focuses on the beliefs of the pharmacy patient and their motivation to contact pharmacies and directly addresses the role communication science plays in pharmacy patients’ interactions in ATCs. 

To examine communication processes, we will refer to pharmacy patients as “subjects”. Kruglanski [10] states that subjects’ desire for information or knowledge leads to NFC and is related to any particular belief properties. These properties may be content-related, structural, a novelty, desirable or formal features in given circumstances. Subjects generate theories that view their own attributes as more predictive of desirable outcomes, and they are reluctant to believe in theories relating their own attributes to undesirable events [12]. These desirable outcomes seem to be explained best by motivational ends according to Kunda [12], or otherwise stated the desire for information or knowledge is a driving motivation. The desire to seek information serves as an example of a motivational force. Motivational forces do not completely blind people to undesirable evidence or information; however, motivational forces could lead people to play down negative information. It should be noted, people’s tendency to link their attributes to desirable outcomes was found only for people who cared about the outcomes. Furthermore, people threatened by undesirable evidence are reluctant to believe this evidence. The desire to seek information is tainted by self-protective motivational forces [12]. Otherwise stated, subjects are satisfied with their current state of knowledge, and do not seek information. 

The framework of attribute satisfaction by consumers has been examined. Attribute satisfaction, then, is the consumer’s subjective satisfaction judgment resulting from observations of attribute performance (role or event) and can be the fulfillment response consumers make when assessing performance [13]. In his research Oliver [13] looked at the role of events (e.g., attribute performance experiences) as causal agents for positive states. This analogy is extended to the summary attribute level, where the sum of positive product experiences (i.e., satisfactory attribute performances) should relate to positive affect, and negative experiences (i.e., dissatisfactory attribute performances) to negative affect [13]. If consumer’s posses dissatisfactory attribute performances and negative outcomes, will they seek information to affect uncertainty?

Cosby and Stephens [14] examined stimulus influences on satisfaction decisions in a study model related to services by an entity. The communication stimuli enhanced satisfaction to services. Subjects given valid information about the services reduced search and evaluation of alternate services within the model. Henceforth in the interpersonal communication process this level of enhanced satisfaction lead to declining levels of uncertainty, with corresponding decreasing information seeking behavior [10]. 

In our examination, the content of the belief properties or attributes of the ATC are general satisfaction, and relevance of information. In this case, information seeking or desire for exchange of information means calling the pharmacy. Therefore, we hypothesize the following about the subjects’ belief properties toward the ATC from pharmacies.

**Hypothesis** **1.** *If subjects are dissatisfied with the content of the ATC received from a pharmacy, then they will seek information*.

Pyszczynski and Greenberg [15] conducted a study to determine causal perceptions of relevant information by subjects, including selection of information. Subjects observed an individual’s behavior in a scenario. Then subjects read personality-related answers provided by the individual in the scenario. A few relevant items help explain the just-observed scenario, and the other items were irrelevant to the scenario. Next the subjects were asked something about the personality of the individual in the scenario. This study design measures the subject’s motivation to voluntarily select information for use in analyzing and providing the answer to the question about the personality of the individual in the scenario. The findings revealed that when faced with disconfirming expectations subjects will seek attribution-relevant information [15]. This research demonstrated when people are confused they search for relevant information. Likewise, the relevance of the information is related to information seeking. This leads to the following hypothesis.

**Hypothesis** **2.** *If subjects feel that the content of the ATC received from a pharmacy is relevant, then they will seek information*.

In addition, communication quality is another belief property associated with the ATC. Webster and Kruglanski [16] conducted an experiment comparing subjective certainty and susceptibility to persuasion in people with different levels of the need for closure. The experimental design was accomplished by introducing participants to differing amounts and quality of information about a situation. The investigators concluded that people with a high NFC are susceptible to the persuasion of differing quality of information, because each persuasive message gives them a chance to achieve closure [16]. This leads us to the third hypothesis, which is as follows.

**Hypothesis** **3a.** *If a subject perceives that the quality of the ATC received from a pharmacy is high, the subject will seek information*.

Likewise, a pre-existing knowledge structure can serve as a motivating factor for search of information. If a subject has both a high affinity for receiving information, and high perception of quality, then we theorized the following:

**Hypothesis** **3b.** *If a subject perceives that the quality of ATC received from a pharmacy is high, and like receiving the ATC, the subject will seek information*.

The extant literature on NFC, indicates the higher the need for closure, the greater the information seeking. Alternatively, a summarization of another finding by Kruglanski [8], states that if the subjects’ confidence in belief properties rank high, along with a high NFC, the tendency to seek information is decreased. If the initial confidence in belief properties ranks low and NFC is high, subjects possess an increased tendency for information seeking. In examining the properties of ATC communication, we decided to test the basic premise of NFC, which leads to the following hypothesis.

**Hypothesis** **4a.** *If subjects, NFC are high after receipt of the ATC from a pharmacy, the subjects will seek information*.

The interaction of variables surfaced from findings in an experiment [17] examining NFC effects and dependent variables to engage informational search by subjects, a multivariate analysis of variance (MANOVA) yielded a significant interaction among variables or belief properties. This unpublished study used a 2 × 2 factorial analysis with two independent and dependent variables to examine interactive effects of need-for-closure. The independent variables were need-for-closure and the subjects’ confidence in the process. The two dependent variables were measures of the subjects’ tendency to engage in information search. The analysis of data showed the two-way interactions were significant (*p* < 0.01) [17]. We chose to examine subjects’ NFC based on preexisting conditions. In particular, the preexisting condition consists of whether patients receiving the ATC from pharmacies like or dislike of receiving the ATC from pharmacies. Besides generally testing NFC, we will test whether people who do not have an affinity for receiving the ATC and have a high NFC would call the pharmacy. These would most likely be the people that we mentioned earlier who call because they find ATCs a bother or nuisance. This leads us to the following sub-hypotheses.

**Hypothesis** **4b.** *If a subject perceives that the quality of the ATC received from a pharmacy is low, and has a high NFC, the subject will seek information*.

Theses related aspects of attribute beliefs found in satisfaction, relevance, and quality along with the theory of need for closure within the theory of uncertainty as embodied in the concept of Interpersonal Communication were analyzed in a random population. We chose validated constructs scales of Satisfaction (Generalized), Information Relevance, Communication Quality and NFC to examine the pharmacy subjects’ response to engaging in the interpersonal communication process. See Table 1 for a summary of the hypotheses. 

## 2. Materials and Methods 

A 46-item questionnaire was compiled as a tool for the survey. Refer to Supplementary Materials for the complete survey. The questionnaire contains six validated construct scales. Validated scales have been tested with successful results many times. Validated construct scales are both reliable and reproducible. The psychometric qualities of each validated construct scales have been provided to verify the validity and reliability of each measure, giving rise to testable theories. This compilation method was utilized to collect data for analysis and inclusion in the 46-item questionnaire survey tool. 

Google survey served as the platform for the survey design, and response collection. The survey was titled “Automated Telephone Calls from Pharmacies” for administration to subjects. The survey design consisted of three sections, Section 1—Pre-Interactions, Section 2—Post-interactions and Section 3—Demographics. Questions in Section 1 of the survey served to separate subjects. If subjects indicated that they have never received prescription medication or never received an ATC from a pharmacy, they were directed to Section 3 of the survey for collection of demographic data. Also, subjects were directed to Section 3 of the survey if they had never received ATCs from a pharmacy. Completion of Section 2 was limited to subjects meeting the criteria set forth in question 1 and/or 2 of Section 1. Only subjects who completed Section 2 responded to all 46-items of the survey. The completion of Section 3 of the survey was required for all subjects in fulfillment of survey completion. The estimated time for completion of the survey was 10-min. The survey was administered using Amazon Mechanical Turk (MTurk) for Social/Behavioral Research projects. MTurk is a web service that provides on-demand scalable human subjects to complete surveys. Keywords, a phrase and a short description were used to assist and guide subjects to participate in the “Automated Telephone Calls from Pharmacies” survey. The keywords are listed in Table 2. 

To aid in the recruitment of subjects, potential subjects were given the following short description concerning the study:

This is a research study that directly addresses automated pharmacy telephone calls and pharmacy customers’ interactions. The purpose of this study is to examine the communication behaviors between pharmacy customers in receipt of automated telephone calls.

After subjects committed to completing the survey, they were directed to complete Section 1. Subjects who met the screening criteria were then directed to Section 2, and then they were directed to indicate their responses and preferences on the validated construct scales. Finally, subjects were directed to Section 3 to report their demographic information. The third section of the survey utilized identical socio-demographic ranges as reported in the United States census. Human subjects 18 years of age or older were eligible participants. The survey was administered using stratified sampling, in six intervals based on age to ensure representation over a continuum, see Table 3 for interval ranges. All subjects were paid for participation. 

### Sources of Validated Construct Scales

The first construct scale to be presented is the “Satisfaction (Generalized)” [18], represented by questions 6 through 10. It is a multi-item, seven-point semantic differential summated ratings scale measuring degree of satisfaction with stimuli. Scale reliability for conducted studies, as measured by Cronbach’s alpha were reported as 0.96, and in other studies 0.94, 0.91, 0.90, 0.93 and 0.87. The Satisfaction scale examines a subject’s degree of satisfaction after receiving automated telephone calls from a pharmacy. High scores indicate greater satisfaction with the automated telephone message, whereas low scores imply that the subjects are not pleased.

The second construct scale to be presented is Information Relevance [19], represented by questions 11 through 15. It is a five-item, seven-point summated rating scale, measuring the level of usefulness a person reports some piece of information to have. Cronbach’s alpha for the Information Relevance scale were reported as 0.94, 0.94, and 0.96. The Information Relevance scale examines the level of usefulness subjects report, concerning the information provided in the automated telephone call. High scores indicate that subjects describe information related to automated telephone calls as being very relevant, whereas low scores imply that the subjects found the information less relevant.

The third construct scale to be presented is Communication Quality [20], represented by questions 16 through 20. It is a five-item, five-point semantic differential scale to assess person’s perceptions of the quality of communication between them and the information provider. For reliability, Cronbach’s alpha for the conducted study was 0.92. The scale examines subjects’ perception of the quality of communication between themselves and the automated telephone message. This Communication Quality scale was reversed scaled. Lower scores on the scale indicate that subjects perceived that high-quality communication occurred between themselves and the automated telephone message, whereas high scores imply that the subjects perceived that low-quality communication occurred between themselves and the automated telephone message.

The forth scale to be presented is a measure of Socially Desirable Response Set (SDRS-5) [21], represented by questions 21 through 25. It is a five-item, five-point scale to evaluate susceptibility to response bias by subjects receiving automated telephone calls from pharmacies. Cronbach’s alpha for the conducted study was 0.66 and 0.68. The scale evaluates a respondent’s tendency to give socially-desirable response[s] [20]. This scale was used to verify that subjects were giving their true response and not giving responses that they thought were socially appropriate.

Finally, the Need for Cognitive Closure scale [22] measured subjects’ tolerance or lack of tolerance for uncertainty. The Need for Cognitive Closure scale consists of 15 items, represented by questions 26 through 40. It consists of a six-point rating scale to measure a variable desire for closure along a continuum with a strong need to attain closure on one end and a high need to avoid closure at the other end [22]. Scale reliability, as measured by Cronbach’s alpha was 0.79. 

## 3. Results

There were 319 respondents who participated in the survey process. Only 294 of the 319 respondents were selected as subjects. Respondents were eliminated only as a result of the screening process or if they did not provide a response to all the questions. The demographic data collected during the survey process serves to provide characteristics of the study population. A brief overview of subject characteristics follows. The female to male ratio was approximately 2:1 respectively for all subjects. Most respondents reported having attended some college and bachelor’s degree for education level. Very few respondents reported education as high school graduate or G.E.D, trade school or other post-secondary education, or associate degree. The majority of the respondents reported being married, followed by single/never married. A preponderance of respondents reported ethnicity or origin as Caucasian/White. Household income ranged from $15K to less than $150K. The interrelationships amongst the subject characteristics and responses were analyzed based on correlations and linear regression. They are not reported here, except for some comparisons, because there were no significant relationships or interaction patterns observed between survey responses and socio-demographic characteristics. None of the 294 subjects scored high on social desirability, which indicates that subjects were giving their true responses, instead of what they think is socially desirable. 

To test the hypotheses, we used correlations and linear regression. The results of the analyses are shown in Table 4. The critical relationship correlations are reported in Table 4.

Hypothesis 1 has been confirmed. Satisfaction with the message content of automated telephone calls is negatively correlated to the number of subjects’ telephone calls to the pharmacy. Although the correlation is small (*r* = −0.0954), it is significant (*p* ≤ 0.10).

Hypothesis 2 has not been confirmed. Relevance of the automated telephone calls is negatively correlated to the number of subject pharmacy telephone calls to the pharmacy; however, this relationship is not significant. However, when we tested just the subjects who liked to receive ATCs, we found that the level of inverse correlation had increased (*r* = −0.2030) and significant (*p* ≤ 0.01).

Quality of the automated telephone call message is positively correlated to the number of subjects’ telephone calls to the pharmacy. This relationship was both positively correlated (*r* = 0.221) and significant (*p* ≤ 0.01). This relationship was further tested by introducing the indicator variable of whether patients liked receiving ATCs as a moderator to quality. This means that subjects who perceive that the quality of the ATC received from a pharmacy is high will seek information. To confirm Hypothesis 3b, we need to add the interaction term and regress both the quality of the automated telephone call (Q) and the interaction of Q and liking (Like) to receive automated telephone calls—I (Q × Like)—on number of subject pharmacy telephone calls to the pharmacy—contacting pharmacies (CP). The linear model is given below.

CP = β_0_ + β_1_·Q + β_2_·I_Q×Like_, (1)

As indicated in Table 5, although the explained variance as measured by *r*^2^ only accounts for 26% of the explained variance, the regression coefficients were both positive and significant; with Quality remaining significant to the *p* ≤ 0.01 level and interaction of liking to receive automated telephone calls and Quality significant to the *p* ≤ 0.05 level. This means that Quality is positively related to the number of phone calls, and that people who like receiving ATCs play an additional role in the quality relationship. Both Hypotheses 3a and 3b have been confirmed.

To test Hypotheses 4a and 4b, we regressed NFC, the interaction between Q and Like, and the interaction of need for closure (NFC) and not liking (NLike) to receive phone calls on to contacting the pharmacies (CP). Some of the results described in the correlation matrix (Table 4) are counter intuitive. For example, Q is negatively correlated to satisfaction. These results led us to test the interaction with Q and Like, since the interaction between NFC and NLike was significant and highly negatively correlated to satisfaction, we wanted to see if isolating people who like receiving phone calls responded differently in terms of the expected measures and whether it was significant. Said another way, we can test whether the people who were dissatisfied with receiving ATCs were driving the counter intuitive relationships. This resulted in the following linear model.

CP = β_0_ + β_1_·NFC + β_2_·I_Q×Like_ + β_3_·I_NFC×NLike_, (2)

The multiple coefficient of determination (*r*) for the regression was 0.71, *r*^2^ was 0.50, while the adjusted-*r*^2^ was also 0.50; with a standard error of 2.87 over 294 observations. Given a mean square error residual (MSR) of 8.23 and a mean square error regression (MSE) of 811, this yields an F(3, 293) of 98.45, meaning that the regression was significant to *p* ≤ 0.01. The regression yielded the following results, Table 6:

Hypothesis 4a has not been confirmed. In general NFC is negatively related to the number of subjects making telephone calls to the pharmacy. This means that people with a high need for closure are generally less likely to contact their pharmacies. This result lends credence to Kruglanski findings [10] that if the subjects’ confidences in belief properties rank high, along with a high NFC, the tendency to seek information is decreased. Hypotheses 4b has been confirmed—people who have a high NFC, who do not like receiving telephone calls from their pharmacy also are likely to call their pharmacy. 

By splitting the relationships using the interaction terms, we received confirmation that the negative relationship between satisfaction and communication quality was driven by NLike. Since the β_1_ coefficient for NFC remained negative, it is consistent with the overall correlations described in Table 4, this indicates that the regression model is correctly specified. A correctly specified model lends credence that the relationships in the linear regression are credible, and not an artifice of multicollinearity. It is interesting that NFC only plays a role in increasing communication with subjects who do not like to receive ATCs from their pharmacy. See Table 7 for a summary of the hypotheses and results. 

## 4. Discussion

We asked how many times have pharmacy personnel answered a telephone, to respond to communications from an automated system received by a subject? We were not able to answer that question, but we were able to survey subjects and determine their motivation for wishing to communicate with their pharmacy. 

Some of the results revealed the situational impact of information seeking. For example, although subjects who like to receive ATCs find the information significantly relevant (*p* ≤ 0.01), the relationship between contacting pharmacies and relevance was inversely correlated. This could be interpreted to mean that since the information is relevant, subjects have no reason to contact the pharmacy after receiving an ATC. This has no bearing on whether patients would contact their pharmacies if they needed information.

We found that NFC played a role only for patients who least liked receiving ATCs. Since we defined a high call volume, as being an individual who makes twelve or more calls to a pharmacy per year, this would mean that a large number of people make these calls based upon not liking to receive automated messages. Although some portion of the calls may be related to therapeutic questions, we suspect a great many are related to either patients’ confusion about why they received the ATC, or a desire to express their displeasure. These incidences of communication would be less productive than addressing patient care needs.

We are less certain about the alternative—the patients who call as a result of having an affinity to receive ATCs. Since there is an interaction between liking the ATC and Quality, there is a need to address why these patients like receiving ATCs. Do these patients just like the aspect of communicating, or do they appreciate the information and seek further guidance and clarity? If it is the later condition of seeking guidance or clarity, then either the message needs to be refined or this follow-up call provided the opportunity to engage in meaningful communication that contributes to better patient care.

What we have found is consistent with other work in human communication research. Kellermann and Reynolds [23] found that while a low tolerance for uncertainty motivates greater information seeking, it is in the negative context. If there is a high level of affinity, then people find it easier to conduct communication. This affinity is a form of attractiveness. Only in the case where there is a high incentive or importance will there be communication under all conditions. This means that patients do not necessarily contact pharmacies based upon whether they are uncertain about the information. Rather, they will contact the pharmacy if they already have a positive relationship or they have a low tolerance for uncertainty. This in-turns means that information sharing will not occur; unless patients are comfortable with the pharmacy, or they have a clear understanding of the need or importance of sharing information.

Readers should notice that both interaction terms are positive and significant. In one case, subjects who like to receive ATCs from pharmacies and who have an affinity for receiving telephone calls were more likely to seek further information. In the other case, people who have a high need for closure, who do not like receiving telephone calls from their pharmacy also were likely to call their pharmacy. This is most interesting, since it indicates that communication is a complex multidimensional concept. Further these findings could possibly be explained by Webster and Kruglanski experimental situation model [18]. If the attribute of attractiveness to the task in the model decreased, subjects reflected a high NFC. We saw that the subjects who did not like receiving ATCs from pharmacies, or found ATCs from pharmacies unattractive possessed a high NFC, and called the pharmacy. 

Although there needs to be further study and refinement on patient communication, one thing our study makes clear is that interpersonal communication is a complex process. The present results open new challenges for this research area. Our findings are consistent with what Kellermann and Reynolds [23] found. By extension, people are more likely to share information if they are comfortable with their provider, or if they already are aware of the importance of the message. This means that ATCs can only serve a limited role. Sending patients a reminder message to pick-up medication or get a flu vaccine via an ATC only works, if they already like the pharmacy or feel that these things are important to them. Otherwise, patients will only contact their pharmacy if they have a low tolerance for uncertainty. They do not contact their pharmacy to avoid uncertainty.

## 5. Conclusions

We were able to identify sub-sets within the study population where either communications and/or interpersonal communication occurred. The contributing factors differed for both groups. The less satisfied subjects—who scored high on the NFC scale—were with the ATC medium, the more likely they were to contact the pharmacy. On the other end of the spectrum, subjects that liked receiving ATCs and perceive the quality of the ATC as high were more likely to contact the pharmacy. Both of these groups of subjects sought to further engage in the interpersonal communication process. 

We do not know how often subjects specifically call pharmacies after receiving ATC. We were unable to determine the degree to which subjects responded to telephone calls because of the limited response options. Question #4 of the survey asked subjects how many times they received calls from pharmacies, while question #5 asked subjects how many times subjects contacted the pharmacy. These scales were less refined. Only three response options were provided for both questions #4 and #5. We recommend refining the number of response options within these questions in future studies, specifically at the lower range. A more refined scale providing seven anchors would yield more precise results. We can only generalize how subjects with a high propensity to contact their pharmacies after receiving an ATC from pharmacies respond. No further interpretation can be made from the study design. Specifically, a more refined scale is important in the formation of a nuanced understanding of the interpersonal communication process. 

We believe that by having a better understanding of patients’ communication traits or beliefs and their receptivity to communication will allow senders to design better mechanisms to interact with patients. To arrive at better mechanisms, further study is needed on why some people (a) do not like receiving ATCs, or (b) what attributes of the ATC interact with some peoples’ innate need for closure. Minor changes to the form or substance of the communication may make an impact on how pharmacies can better utilize forms of communication. Having an understanding of how and when patients wish to receive communication is critical to designing better communication mechanisms. Accordingly, Sileo and Kayson [24] found that the time of day can affect responsiveness to messaging. Care must be taken to insure that healthcare providers consider patients’ receptivity to communication, not just the message itself. As illustrated by Xu, Bates and Schweitzer [25]—when examining telephone messaging in facilitating communications, they were unable to find a significant difference among specific message types. Extensions to this research will allow practitioners to improve the communication process, by either changing ATC messages or segmenting patients, or some combination of changing ATC messages and segmenting patients and then tailoring the message according to patient segments.

## Figures and Tables

**Table 1:** Summary of hypotheses for phenomena of behaviors of pharmacy patients in receipt of an automated telephone call (ATC).

Item	Hypothesis
1.	If subjects are dissatisfied with the content of the ATC received from a pharmacy, then they will seek information.
2.	If subjects feel that the content of the ATC received from a pharmacy is relevant, then they will seek information.
3a.	If a subject perceives that the quality of the ATC received from a pharmacy is high, the subject will seek information.
3b.	If a subject perceives that the quality of ATC received from a pharmacy is high, and like receiving the ATC, the subject will seek information.
4a.	If subjects, NFC are high after receipt of the ATC from a pharmacy, the subjects will seek information.
4b.	If a subject perceives that the quality of the ATC received from a pharmacy is low, and has a high NFC, the subject will seek information.

**Table 2:** Keywords to recruit subjects.

Keywords (Alphabetical Order):			
Behaviors	Customers	Medicines	Prescriptions
Capsules	Healthcare	Pharmacies	Interactions
Communications	Medications	Pills	Tablets

**Table 3:** Intervals of survey administration based on age.

Group	Age Range
1	Ages 18 years < 25 years
2	Ages 25 years < 30 years
3	Ages 30 years < 35 years
4	Ages 35 years < 45 years
5	Ages 45 years < 55 years
6	Ages 55 years or older

**Table 4:** Correlations of critical relationships.

	Satisfaction	Relevance	Quality	SDRS	NFC	I (Q × Like)	I (NFC × Not Like)	Contacted
Satisfaction	1							
Relevance	0.8264	1						
Quality	−0.6760	−0.6981	1					
SDRS	0.0526	0.0478	−0.0341	1				
NFC	0.1574	0.1439	−0.1997	−0.1173	1			
I (Q × Like)	0.4751	0.3631	−0.1200	−0.0420	0.0388	1		
I (NFC × not like)	−0.7663	−0.6348	0.4945	0.0089	0.0022	−0.7972	1	
Contacted	−0.0954	−0.0845	0.2210	0.0214	−0.0431	0.1082	0.0298	1

**Table 5:** Regression results communication quality.

Variable	*β_i_*	Std. Error	*t* Stat	*p*
Intercept	0.8473	0.0637	13.3034	≤0.01
Q	0.0234	0.0056	4.1630	≤0.01
I (Q × Like)	0.0138	0.0057	2.3976	≤0.05

**Table 6:** Regression results NFC and interaction terms.

Variable	*β_i_*	Std. Error	*t* Stat	*p*
Intercept	6.6326	0.9998	6.6336	≤0.01
NFC	−0.0795	0.0142	−5.6087	≤0.01
I (Q × Like)	0.7958	0.0702	11.3405	≤0.01
I (NFC × not like)	0.1636	0.0101	16.2671	≤0.01

**Table 7:** Summary of hypotheses and results for phenomena of behaviors of pharmacy patients in receipt of an automated telephone call (ATC).

Item	Hypothesis	Result	Relationship	Significance
1.	If subjects are dissatisfied with the content of the ATC received from a pharmacy, then they will seek information.	Confirmed	*r =* −0.0954 negative as predicted	*p ≤* 0.10
2.	If subjects feel that the content of the ATC received from a pharmacy is relevant, then they will seek information.	Not confirmed	*r* = −0.2030 predicted positive but negative relationship	*p ≤* 0.01
3a.	If a subject perceives that the quality of the ATC received from a pharmacy is high, the subject will seek information.	Confirmed	*r* = 0.221 positive as predicted	*p* ≤ 0.01
3b.	If a subject perceives that the quality of ATC received from a pharmacy is high, and like receiving the ATC, the subject will seek information.	Confirmed	β = 0.0234 positive as predicted	*p* ≤ 0.01
4a.	If subjects, NFC are high after receipt of the ATC from a pharmacy, the subjects will seek information.	Not confirmed	β = −0.0795 predicted positive but negative relationship	*p* ≤ 0.01
4b.	If a subject perceives that the quality of the ATC received from a pharmacy is low, and has a high NFC, the subject will seek information.	Confirmed	β = 0.1636 positive as predicted	*p* ≤ 0.01

## Supplementary Materials

The following are available online at http://www.mdpi.com/2226-4787/6/2/47/s1, Table S1: survey, “Automated Telephone Calls from Pharmacies”, S2: spread sheet, data set.
