# Supplementary material for: Uncertainty and Motivation to Seek Information from Pharmacy Automated Communications
Source: Pharmacy (Basel). 2018 May 28;6(2):47. doi: 10.3390/pharmacy6020047 (PMC6025562; doi:10.3390/pharmacy6020047)
Supplement: Supplementary file 1 [file pharmacy-06-00047-s001.zip › Supplementary Data/Automated Telephone Calls Survey.pdf]

Table 1. Automated Telephone Calls from Pharmacies

*Required
R = reverse scale

Section 1 of 3 Pre-Interaction

1.	**Have you ever had the occasion to use prescriptions medications?*** *If no skip to question 41*	Yes	No	
2.	**Have you ever received automated telephone calls from a pharmacy?*** *If no skip to question 41*	Yes	No	

Section 2 of 3 Post-Interaction

3.	**Do you like receiving automated telephone calls from a pharmacy?*** If no skip to question 41	Yes	No						
4.	**How often do you receive automated telephone calls from a pharmacy?*** *Mark only one.*	4 times or less /year	5 to 11 times/year	12 times or more/year					
5.	**How often do you contacted a pharmacy for clarification of a message received from an automated telephone message?*** *Mark only one.*	4 times or less /year	5 to 11 times/year	12 times or more/year					
		Dissatisfied					Satisfied		
6.	**After receiving an automated phone call from a pharmacy, which of the following best describes your experience?*** *Mark only one.*	1	2	3	4	5	6	7	
		Displeased					Pleased		
7.	**After receiving an automated phone call from a pharmacy, which of the following best describes your experience?*** *Mark only one.*	1	2	3	4	5	6	7	
		Unfavorable					Favorable		
8.	**After receiving an automated phone call from a pharmacy, which of the following best describes your experience?*** *Mark only one.*	1	2	3	4	5	6	7	
		Unpleasant					Pleasant		
9.	**After receiving an automated phone call from a pharmacy, which of the following best describes your experience?*** *Mark only one.*	1	2	3	4	5	6	7	
		I didn't like it at all					I like it very much		
10.	**After receiving an automated phone call from a pharmacy, which of the following best describes your experience?*** *Mark only one.*	1	2	3	4	5	6	7	

Table 1. continued

		Not at all					Very much	
11.	**After receiving automated telephone calls from a pharmacy, how relevant was the information provided?*** *Mark only one.*	1	2	3	4	5	6	7
12.	**After receiving automated telephone calls from a pharmacy, how meaningful was the information provided?*** *Mark only one.*	1	2	3	4	5	6	7
13.	**After receiving automated telephone calls from a pharmacy, how important was the information provided?*** *Mark only one.*	1	2	3	4	5	6	7
14.	**After receiving automated telephone calls from a pharmacy, how useful was the information provided?*** *Mark only one.*	1	2	3	4	5	6	7
15.	**After receiving automated telephone calls from a pharmacy, how helpful was the information provided?*** *Mark only one.*	1	2	3	4	5	6	7
		Untimely				Timely		
16.	**To what extent do you feel that the communication received from the automated telephone message is:*** (R) *Mark only one.*	1	2	3	4	5		
		Inaccurate				Accurate		
17.	**To what extent do you feel that the communication received from the automated telephone message is:*** (R) *Mark only one.*	1	2	3	4	5		
		Inadequate				Adequate		
18.	**To what extent do you feel that the communication received from the automated telephone message is:*** (R) *Mark only one.*	1	2	3	4	5		
		Incomplete				Complete		
19.	**To what extent do you feel that the communication received from the automated telephone message is:*** (R) *Mark only one.*	1	2	3	4	5		
		Not Credible				Credible		
20.	**To what extent do you feel that the communication received from the automated telephone message is:*** (R) *Mark only one.*	1	2	3	4	5		

Table 1. continued

#	Item						
		Definitely false				Definitely true	
21.	**I am always courteous even to people who are disagreeable.*** (R) *Mark only one.*	1	2	3	4	5	
22.	**There have been occasions when I took advantage of someone.*** *Mark only one.*	1	2	3	4	5	
23.	**I sometimes try to get even rather than forgive and forget.*** *Mark only one.*	1	2	3	4	5	
24.	**I sometimes feel resentful when I don't get my way.*** *Mark only one.*	1	2	3	4	5	
25.	**No matter who I'm talking to, I'm always a good listener.*** (R) *Mark only one.*	1	2	3	4	5	
		Completely disagree				Completely agree	
26.	**I don't like situations that are uncertain.*** *Mark only one.*	1	2	3	4	5	6
27.	**I dislike questions which could be answered in many ways.*** *Mark only one.*	1	2	3	4	5	6
28.	**I find that a well ordered life with regular hours suit my temperament.*** *Mark only one.*	1	2	3	4	5	6
29.	**I feel uncomfortable when I don't understand the reason why an event occurred in my life.*** *Mark only one.*	1	2	3	4	5	6
30.	**I feel irritated when one person disagrees with what everyone else in a group believes.*** *Mark only one.*	1	2	3	4	5	6
31.	**I don't like to go into a situation without knowing what I can expect from it.*** *Mark only one.*	1	2	3	4	5	6
32.	**When I have made a decision, I feel relieved.*** *Mark only one.*	1	2	3	4	5	6
33.	**When I am confronted with a problem, I'm dying to reach a solution quickly**.* *Mark only one.*	1	2	3	4	5	6
34.	**I would quickly become impatient and irritated if I would not find a solution to a problem immediately.*** *Mark only one.*	1	2	3	4	5	6
35.	**I don't like to be with people who are capable of unexpected actions.*** *Mark only one.*	1	2	3	4	5	6
36.	**I dislike it when a person's statement could mean many different things.*** *Mark only one.*	1	2	3	4	5	6
37.	**I find that establishing a consistent routine enables me to enjoy life more.*** *Mark only one.*	1	2	3	4	5	6
38.	**I enjoy having a clear and structured mode of life.*** *Mark only one.*	1	2	3	4	5	6
39.	**I do not usually consult many different opinions before forming my own view.*** *Mark only one.*	1	2	3	4	5	6
40.	**I dislike unpredictable situations.*** *Mark only one.*	1	2	3	4	5	6

Table 1. continued

Section 3 of 3 Post-Interaction

41. **Sex*** *Mark only one.*
    Female
    Male
42. **Age*** *Mark only one.*
    18-24 years
    25-29 years
    30-34 years
    35-39 years
    40-44 years
    45-49 years
    50-54 years
    55-59 years
    60-64 years
    65 years or over
43. **Education*** *Mark only one.*
    Less than high school
    High school graduate or General Equivalency Diploma, G.E.D.
    Trade school or other post secondary education
    Some College
    Associate's degree
    Bachelor's degree
    Graduate/Professional degree
44. **Marital Status*** *Mark only one.*
    Single/never married
    Married
    Divorced/separated
    Widow
    Domestic partner
    Member of an unmarried couple
45. **Ethnicity or Origin** * *Check all that apply.*
    African-American/Black
    American Indian, Eskimo or Aleut
    Asian Pacific Islander
    Caucasian/White
    Hispanic or Latino
    Native Hawaiian or other Pacific Islander
    Multiracial
    Prefer not to answer
    Other:__________________
46. **Annual Household Income*** *Mark only one.*
    Less than $15K
    $15 K to less than $25 K
    $25 K to less than $35 K
    $35 K to less than $50 K
    $50 K to less than $75 K
    $75 K to less than $100 K
    $100 K to less than $150 K
    $150 K to less than $200 K
    $200 K or higher
